# Chronic Hydrocephalus

**Published:** 1871-01

**Authors:** 


### Clinical Reports.

### CHRONIC HYDROCEPHALUS.

Clinic by Prof. N. S. DAVIS. Reported by F. H. D.

MERCY HOSPITAL, *December 8, 1870.*

This little child has been brought in for your inspection to-day, as presenting a very good example of chronic hydrocephalus or dropsy of the brain. The entire head, you perceive, is enlarged to nearly twice the normal size, the bones being widely separated at the sutures. The occipital bone forms a firm fixed base, while the upper part of the cranium, yielding easily at the ununited sutures, gradually enlarges as the effusion increases. There is a peculiar bulging appearance of the eyes, caused by the pushing forward and downward of the frontal bone, decreasing the orbital space, thus crowding the eyeball outward and turning the axis of the eyes somewhat downward. The scalp is quite tense over the separated sutures, and the fluctuation very distinct upon pressure. The general nutrition of the body and limbs, is better than is usual in these cases.

This child is now about a year old. Its head was first noticed to be enlarging after an attack of fever, accompanied by derangement of the stomach and bowels, which occurred when it was three months old. The enlargement had been progressing about a month when the case was first brought to my office. From the rapid rate at which the disease was progressing, I judged that the child would live but a short time. It was, however, placed under treatment, and seen several times at short intervals. I then lost all track of the case, and supposed the child had died. A few days ago, however, it was again brought to me in the condition in which you now see it. During the interval since I had seen it last, the mother stated that it had been kept constantly upon the treatment that I had directed last. The size of the head has increased some, but the enlargment has not been so rapid or extensive as is usual in these cases.

The cases of chronic hydrocephalus may be distinguished into three classes. The first class includes the cases in which the disease is congenital. The child, in these cases, usually lives but a few days, although life may occasionally be prolonged for several months.

In the second class of cases, the child is apparently healthy at birth, and continues so until between the second and third months. It is then attacked with acute symptoms of restlessness and crying out in sleep. The discharges from the bowels are variable in character, frequently of a greenish color. Vomiting is frequent if the child is raised up. These symptoms continue for two or three days, when there supervenes an attack of convulsions, and the child will now be found to be laboring under all the symptoms of effusion on the brain. Previous to the occurrence of the convulsions, the pupils are usually contracted; after their occurrence, however, the pupils will be found to be widely dilated, and a want of co-ordination will be noticed in the eyes, the axis being turned in different directions. There may be but a single attack of convulsions, or they may be repeated several times. Some cases terminate fatally in a very short time; the fever increasing, the pupils becoming more widely dilated, the face pale, and coma and death supervening.

In young children, where the sutures are still ununited, the head easily expands as the exudation increases. The inflammatory stage and active symptoms then pass off; some restlessness and starting in sleep continues. The child cannot hold up or steady the head well. The muscles of the extremities are also weak, so that it cannot stand. The parents may, very likely, suppose for four or five weeks, that the child is improving and getting well, considering the debility and muscular weakness as merely the efiects of the previous disease, but as time passes on the head is observed to be enlarging, the urine is scanty and the bowels deranged, passages variable in character. In two or three months the head will sometimes have attained the size of this one.

The third class, of which the case before you is a very good example, are of more obscure origin. There are no active symptoms to commence with. Slight fever and restlessness, the stomach and bowels deranged, and urine scanty as in the other cases. The child acts as if in pain every time it is moved; no convulsions, however, supervene. In six to ten days the fever disappears, and only slight derangement of stomach and bowels remains, the food frequently passing through undigested—occasionally the bowels are constipated. In this way they will continue for perhaps a month, when it is noticed that the head is enlarging and the child is growing weaker. It cannot hold up the head, and is averse to the upright position. The surface generally is pale, the veins passing over the head are enlarged and blue. When the head is examined the sutures are found to be wide open. The intelligence is apparently unimpaired, there is generally considerable weakness of the extremities, so that it cannot stand, or attempt much use of his legs.

The pathology of this disease, except in the congenital cases, seems to consist of a low grade of inflammation in the pia-mater and arachnoid membranes. Hereditary tubercle frequently forms the starting point of the disease. When not traceable to this tuburculous origin, it usually originates from simple subacute inflammation. The inflammatory stage lasts but a short time. The effusion, however, continues.

The large majority of these cases terminate fatally, although some of the children live for a considerable time. In a case to which I directed the attention of the class some years ago, the child had lived to be four years old. The head had expanded until it had become heavier than all the rest of the body, and the child could not be supported in an upright position for even an instant without fainting. Another case, about the same age as this one, was brought before the class last winter, and was placed under treatment, although without any expectation of a favorable result. It soon began, however, to improve, and continued to progress favorably for three months, when I lost sight of the child, and do not know whether it finally recovered or not.

*Treatment.*—The books give no encouragement for the treatment of these cases. There are two methods, however, that have been proposed, the one surgical, the other medical. The late Prof. Brainard was one of the first, I believe, to attempt the cure of these cases by the insertion of a small trochar, allowing a little of the fluid to escape, and then injecting a weak solution of iodine and iod. of potass. into the cavity. The theory was, that the contact of the iod. with the surface of membranes would stop further effusion. Some considerable disturbance, nervous twitching, etc., were produced at the time of the injection. These symptoms passed away in a short time, and no further effects were manifest from it. In one case, I think, he repeated the injection as many as six or seven times. No case of successful result from this operation has, however, been recorded.

The objects that I have attempted to accomplish by treatment in these cases has been, first, to allay the morbid excitement of the cerebral structures, and, second, to exert a gentle, persistent. and long-continued alterative and diuretic influence, avoiding carefully any impairment of the digestive organs. I have succeeded in accomplishing these purposes by the following prescription:

℞ Fl. ex. scutellaria ℥ ii.

Tinct. digitalis, ℥ ss.

Iod. potass., ʒ ij.

Fl. ex. hyosciamus, ℥ ss.

M.

Dose, 20 drops; four times a day, in sweetened water.

If the digitalis is found to be exerting too much influence, the dose must be diminished.

Mercurials are of no advantage in the chronic stage. During the early inflammatory stage, mercurials, combined with mild laxatives, might check the progress of the disease; and if promptly followed by efficient doses of the iodide of potassa, any considerable effusion would be prevented, except in such cases as are complicated with tubercular deposits. If effusion does take place, and the case becomes chronic, it will be better to unite the iodide with the digitalis, scutellaria, etc., as in the formula already given to you.

